# Epidemiology of the First Wave of COVID-19 ICU Admissions in South Wales—The Interplay Between Ethnicity and Deprivation

**DOI:** 10.3389/fmed.2020.569714

**Published:** 2020-10-07

**Authors:** Thomas Baumer, Emily Phillips, Amrit Dhadda, Tamas Szakmany

**Affiliations:** ^1^Department of Anaesthesia, Royal Gwent Hospital, Newport, United Kingdom; ^2^Department of Critical Care, Royal Gwent Hospital, Newport, United Kingdom; ^3^Department of Anaesthesia, Intensive Care and Pain Medicine, Division of Population Medicine, Cardiff University, Cardiff, United Kingdom

**Keywords:** COVID-19, ethnicity, BAME, deprivation, mortality

## Abstract

On the 9th March 2020, the first patient with COVID-19 was admitted to ICU in the Royal Gwent Hospital (RGH), Newport, Wales. We prospectively recorded the rate of ICU admissions of 52 patients with COVID-19 over 60 days, focusing on the epidemiology of ethnicity and deprivation because these factors have emerged as significant risk factors. Patients were 65% (34 of 52) male and had a median (IQR) age of 55 (48–62) years. Prevalent comorbidities included obesity (52%); diabetes (33%), and asthma (23%). COVID-19 hospital and ICU inpatient numbers peaked on days 23 and 39, respectively—a lag of 16 days. The ICU mortality rate was 33% (17 of 52). People of black, Asian, and minority ethnic descent (BAME group) represented 35% of ICU COVID-19 admissions (18 of 52) and 35% of deaths (6 of 17). Amongst the BAME group, 72% (13 of 18) of patients were found to reside in geographical areas representing the 20% most deprived in Wales, vs. 27% of patients in the Caucasian group (9 of 33). Less than 5% of the population within the area covered by RGH are of BAME descent, yet this group had a disproportionately high ICU admission and mortality rate from COVID-19. The interplay between ethnicity and deprivation, which is complex, may be a factor in our findings. This in turn could be related to an increased prevalence of co-morbidities; higher community exposure; larger proportion of lower band key worker roles; or genetic polymorphisms.

## Introduction

Severe acute respiratory syndrome-coronavirus-2 (SARS-CoV-2) infection, originating from Wuhan, China is the underlying cause of coronavirus disease-2019 (COVID-19) (1). The pandemic reached Italy in February and caused significant strain on the Lombardy critical care units (2). The first known patient with SARS-CoV-2 infection was discovered in Wales on the 2nd March 2020. The first COVID-19 patient admitted to an ICU in Wales was admitted to the Royal Gwent Hospital (RGH) in Newport on the 9th March. Heeding the warning from Italy, the RGH moved early to cancel elective activity and prepare for a significant increase in admissions (3).

The RGH in Aneurin Bevan University Health Board (ABUHB) is the major specialist center, which provides secondary services to a population of ~400,000 people. The overall geographical region from which RGH's acute admissions are received, known as the catchment area, includes a higher number of deprived neighborhoods than any other hospital in Wales according to the 2011 Census data from the UK Office of National Statistics (ONS).

We have previously reported that social deprivation is an independent risk factor of long-term outcome following critical care discharge (4). Social deprivation is often linked to reduced access to healthcare and this has been a particular problem amongst the Black, Asian and Minority Ethnic (BAME) population group. The acronym “BAME” is used for research purposes in the UK and includes people not of Caucasian ethnicity. BAME is an umbrella term, which the UK census reports as four broad subgroups: black/black British, Asian/Asian British, mixed-race, and other non-Caucasian minority ethnic people.

During the first wave of the COVID-19 pandemic, data emerged indicating that people of BAME descent in the UK are at higher risk of intensive care unit (ICU) admission and death with COVID-19 (5). In this case-series analysis we report the epidemiology of the first wave of COVID-19 patients in South-East Wales admitted into ICU, and describe the connection between ethnicity and social deprivation using data from the first 60 days of the COVID-19 pandemic in the RGH.

## Methods

### Conceptualization and Approval

Our prospective service evaluation on the ICU was developed before the first patient with COVID-19 was admitted. The ABUHB Risk Review Committee approved the project and waived the need for written informed consent. All data collected was fully anonymised and summarized as an absolute number (with percentage), or as a median value (with interquartile range), as appropriate.

### Setting

RGH is a medium-size district general hospital with 800 inpatient beds in Newport, South Wales. The critical care unit is normally a 16-bedded combined ICU (maximum of 12 beds for invasive ventilation with 1:1 nurse:patient ratios) and high-dependency unit (for patients needing other organ support, including non-invasive ventilation with 1:2 nurse:patient ratios) located in two areas on the same floor. We have previously described the case-mix and our approach to flexibly use the critical care resources (6).

During the first-wave of COVID-19 admissions, the critical care capacity has been upscaled to a 40 bedded ICU spanning three separate areas with increased nurse-to-patient ratios (3). All patients with suspected or confirmed COVID-19 disease were enrolled in a multicenter clinical trial as appropriate (7, 8). We have not used any novel disease modifying agents or therapies outside these clinical trials.

### Clinical Data Sources

Data on COVID-19 patients admitted to the ICU was collected prospectively from 9th March 2020 (day 1) to the 7th May 2020 (day 60). COVID-19 was defined as confirmed (respiratory failure and radiological changes with SARS-CoV-2 RNA detected on PCR testing), or suspected (respiratory failure and radiological changes consistent with COVID-19 but without a confirmed PCR test result). We recorded patient age; sex; BMI; need for any assistance with daily activities; APACHE II score; time from hospital admission to ICU, and ICU mortality.

### Epidemiological Data Sources

To put the ICU admissions into context and to aid in national comparisons, we utilized the daily situation report data from the Welsh Government Acute Secondary Care COVID-19 Group, available from the 23rd March 2020 (day 16) onwards. This administrative data summarized the daily patient admissions and discharges, including the COVID-19 related deaths reported in each Welsh hospital.

We recorded ethnicity for each patient in the study according their Caucasian or BAME subgroup status, while background data on ethnicity within RGH catchment area was obtained from the ONS 2011 census data. We used patients' postcodes in order to place them into their respective census neighborhood localities - known as “lower super output areas” (LSOAs). In census terms, LSOAs are geospatial statistical units possessing a similar population size, and homogeneity regarding tenure of households and dwelling type.

Deprivation within each LSOA was ranked using the Welsh Index of Multiple Deprivation (WIMD) quintiles from the ONS. Patients' postcodes (assumed to be a surrogate marker of deprivation) and ethnicity were plotted in their corresponding LSOAs using the interactive online WIMD map tool at https://wimd.gov.wales/.

## Results

### Overview

**Figure 1:**
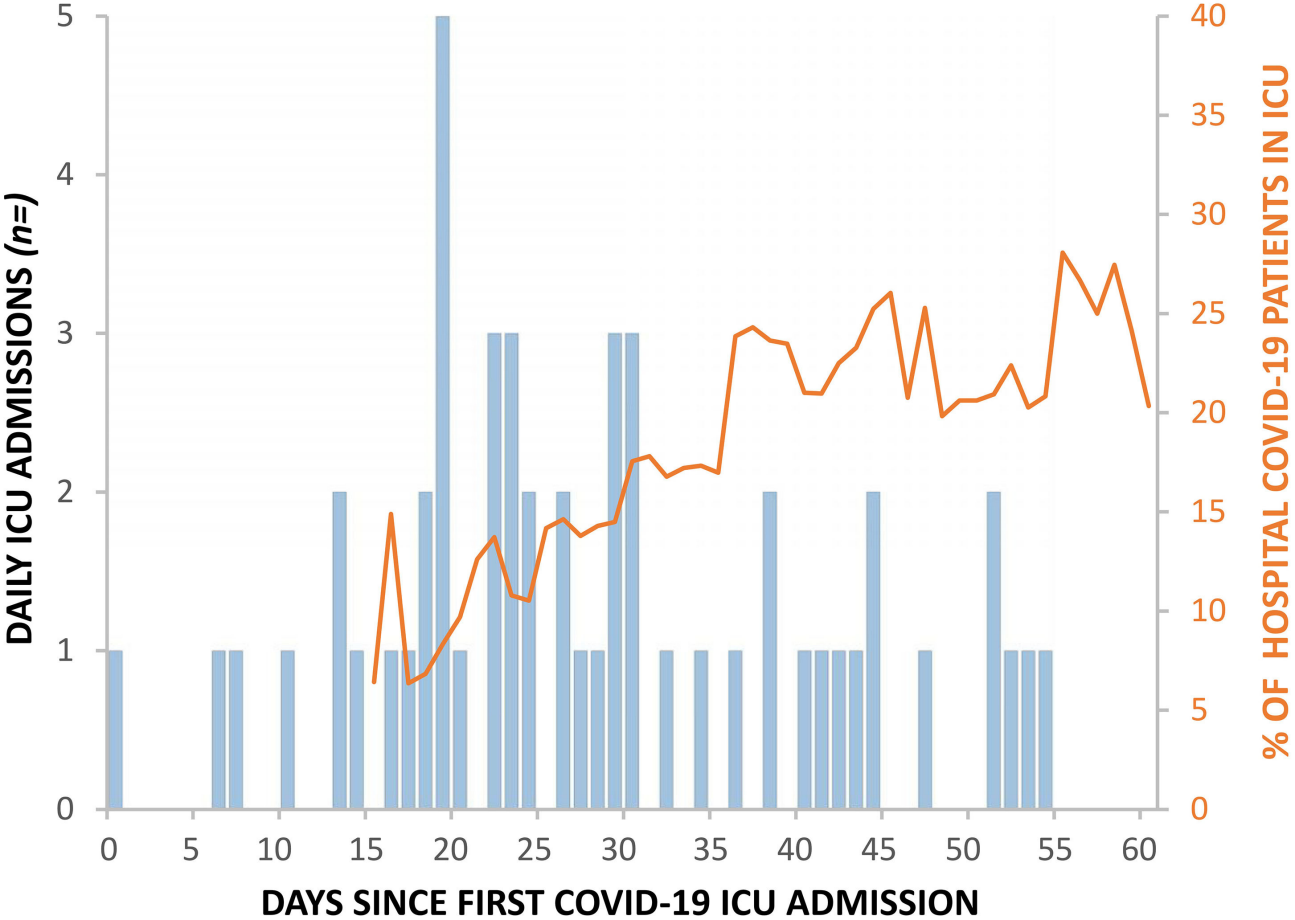
Daily ICU COVID-19 admissions (blue bars) alongside corresponding percentage of total hospital COVID-19 inpatients in ICU (orange line).

Between 9th March 2020 (day 1) and 7th May (day 60), 52 patients (46 confirmed and 6 suspected) with COVID-19 disease were admitted to the ICU. All patients were mechanically ventilated on admission. Table 1 describes the demographics of the 52 patients admitted to the ICU. One out of 52 patients needed assistance with daily activities of living before admission. Figure 1 shows the daily ICU admission figures from 9th March and their relation to the total number of patients in the hospital with COVID-19 disease from 23rd March onwards (day 15).

**Table 1:** Demographics of COVID-19 ICU patients.

	**Patients (*n* = 52)**
Age; years	55 (48–62)
**Gender**	
Female	18 (34.6%)
Male	34 (65.4%)
APACHE II score on admission	12 (10–15)
Hospital admission to ICU time; days	1.32 (0.36–3.66)
**Ethnicity**	
White	34 (65.4%)
Mixed	0 (0.0%)
Asian/Asian British	12 (23.1%)
Black/Black British	3 (5.8%)
Other	3 (5.8%)
**Wales index of multiple deprivation**	
Quintile 1—least	6 (11.5%)
Quintile 2	7 (13.5%)
Quintile 3	11 (21.2%)
Quintile 4	5 (9.6%)
Quintile 5—most	22 (42.3%)
**Common comorbidities**	
Essential hypertension	18 (34.6%)
Diabetes (type 1 and 2)	17 (32.7%)
Asthma (all severities)	12 (23.1)
Ischaemic heart disease	4 (7.7%)
Chronic kidney disease	2 (3.8%)
**BMI**	
18.5– <25	6 (11.5%)
25– <30	17 (32.7%)
30– <40	24 (46.2%)
40+	3 (5.8%)
Unknown	2 (3.8%)

*Values are median (IQR) or number (proportion)*.

### Exceeding Maximum Capacity

**Figure 2:**
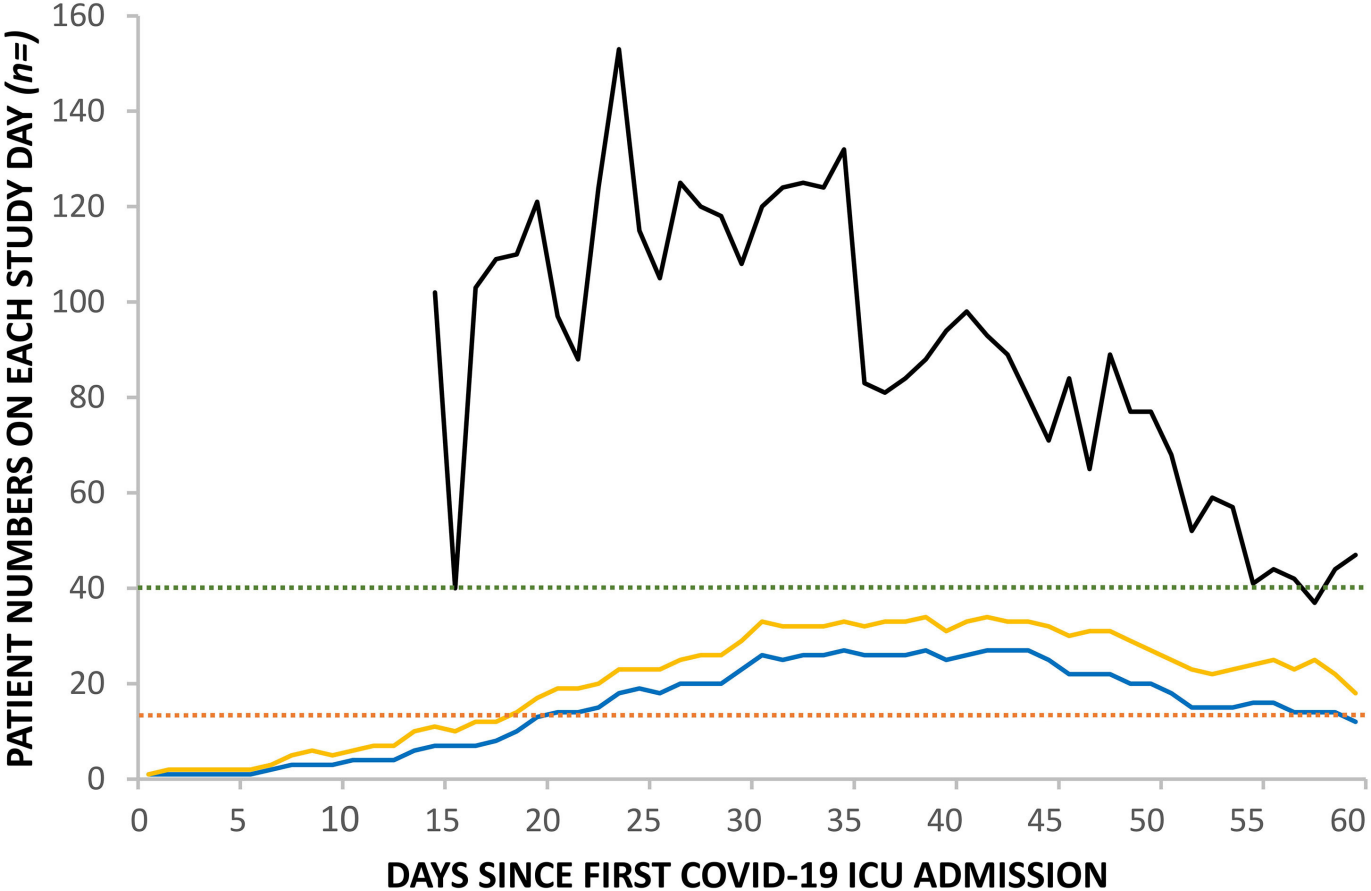
Change in daily inpatient numbers in RGH: COVID-19 ward numbers (black line), COVID-19 ICU numbers (blue line), and total ICU numbers (yellow line). Pre-pandemic ICU maximum capacity for invasively ventilated patients (red dashed line); upscaled pandemic maximum capacity for invasively ventilated patients (green dashed line).

**Figure 3:**
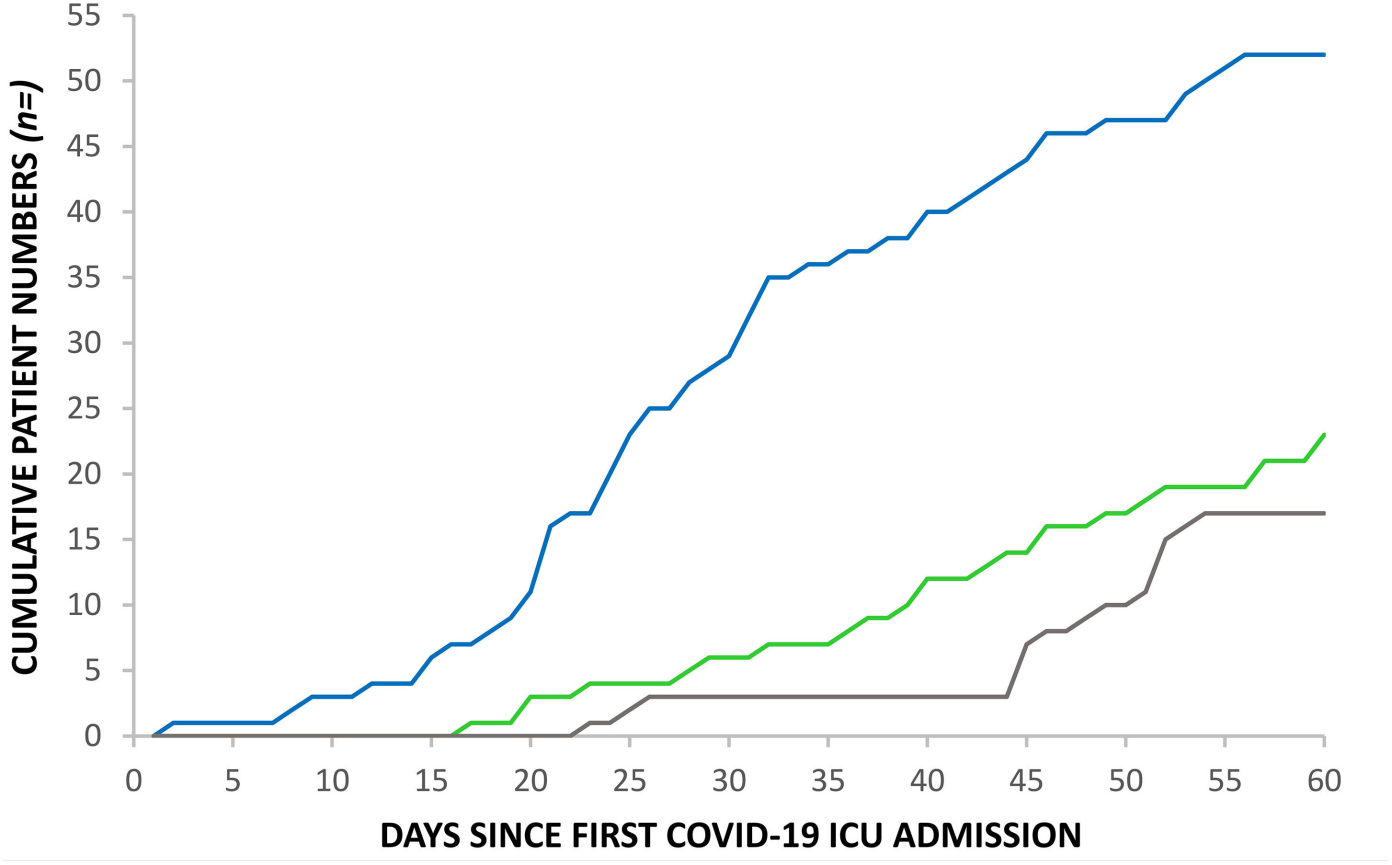
Cumulative admissions, discharges and deaths to RGH ICU: COVID-19 ICU admissions (blue line), COVID-19 ICU discharges (green line) and COVID-19 ICU deaths (gray line).

The RGH critical care unit went exceeded normal maximum capacity on the 28th March, 20 days after the first admission with COVID-19 and remained above maximum capacity for 40 days (Figure 2). Ward inpatient numbers peaked on the 31st March (day 23) while ICU numbers peaked, after a 16 day lag, on the 16th April (day 39), when calculated using a seven-day moving mean across the 60 day study. Figure 3 demonstrates how the first patient was discharged alive on the 25th March, 17 days following the first admission with COVID-19, while the first death occurred on the 31st March (day 23).

### Ethnicity, Admissions, and Mortality

We contrasted the admission and death rates of ICU of patients in the Caucasian and BAME subgroups to their local representation in the 2011 census of the RGH catchment area background population (Table 2). Asians/British Asians had the highest ICU admission rate (116.4 per 100,000) and ICU death rate (48.5 per 100,000) of any ethnic group, compared to Caucasians at 8.6 per 100,000 ICU admissions and 2.8 per 100,000 ICU deaths.

**Table 2:** COVID-19 ICU admissions and mortality by ethnicity—Causasian group and BAME subgroups.

	**RGH catchment area—Background population (*n* = 415,617)**	**RGH ICU—Covid-19 patients**	
		**Admissions (*n* = 52)**	**Mortality (*n* = 17)**
Caucasian	396,101 (95.3%)	34 (65.4%)	11 (64.7%)
BAME:	19,516 (4.7%)	18 (34.6%)	6 (35.3%)
Mixed	4,529 (1.1%)	0 (0.0%)	0 (0.0%)
Asian/Asian British	10,309 (2.5%)	12 (23.1%)	5 (29.4%)
Black/Black British	2,964 (0.7%)	3 (5.8%)	1 (5.9%)
Other	1,732 (0.4%)	3 (5.8%)	0 (0.0%)

*Values are numbers (proportion)*.

### Ethnicity and Deprivation

In terms of deprivation, nine out of 33 patients (27%) in the Caucasian group compared to 13 of 18 patients (72%) in the BAME group, lived in areas in the bottom quintile of the WIMD (most deprived). The Asian/British Asian subgroup had the highest proportion, 11 of 13 (85%), within the bottom quintile.

Out of patients in the BAME group, 12 of 18 patients (66%) lived in a multi-generational household; three of them were unemployed, and eight of them were employed in a lower band key-worker role. In contrast, none of the Caucasian patients lived in a multi-generational household; one was a pensioner; one was unemployed, and none worked in key-worker roles.

**Figure 4:**
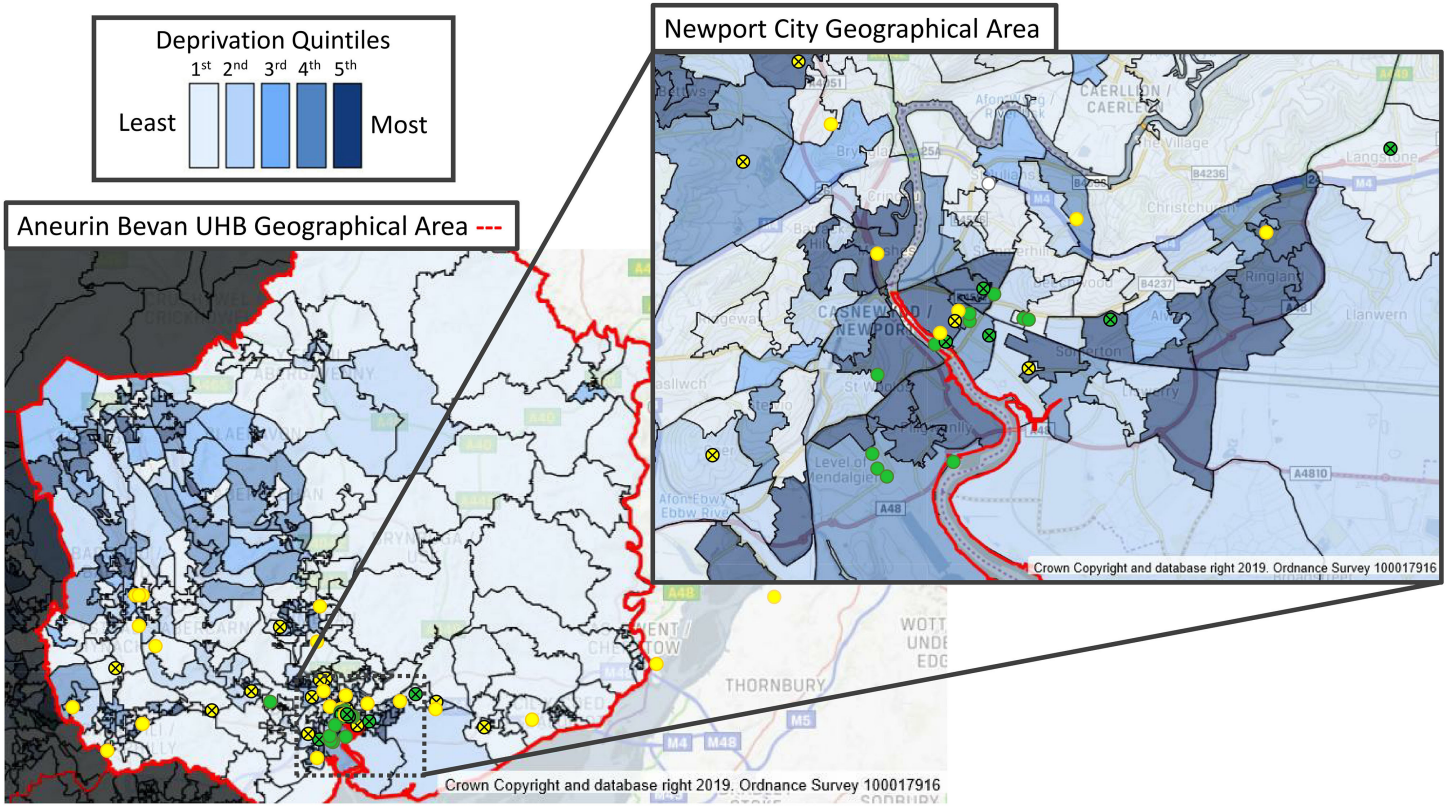
Ethnicity, deprivation and COVID-19 ICU admissions from the RGH ICU catchment area postcodes of COVID-19 ICU individual patients, plotted on a map of “Welsh Index of Multiple Deprivation” (WIMD) quintiles, indicating ethnicity and ICU outcome. Caucasian (yellow circles); BAME (green circles); survivor (open circles); deceased (crossed circles).

The absolute number of deaths was highest in the bottom quintile for both Caucasian and BAME patients. More patients died in the BAME group within the bottom quintile: 4 of 6 deaths vs. 3 of 11 deaths in the Caucasian group. Figure 4 shows the interplay between socioeconomic deprivation, ethnicity and outcome.

## Discussion

During the first wave of the COVID-19 pandemic, we found that patients from the BAME group are at significantly higher risk of ICU admission and death from COVID-19. Although the hospital's catchment area covers an overwhelmingly white ethnic population, over a third of the admissions to the critical care unit were from the BAME group. Notably, we observed a significant interplay between deprivation and ethnicity.

The lag between the peak of hospital admissions and the peak of ICU admissions provides an important buffer and could be used in future modeling of ICU capacity for anticipated further waves of COVID-19 outbreaks. We have further observed a sharp decline in hospital admissions, but a much slower return to normal number of patients cared for on the ICU after the stricter lockdown measures were implemented in Wales. This is in part explained by our long ICU length of stay for both survivors and non-survivors.

As our patient population was relatively young, with fewer comorbidities and frailty than the Welsh average, we have opted for keeping active treatment as long as feasible in a bid to improve outcomes (3, 9). Compared to some large European cohorts, our patients were significantly younger, however this was in line with the national experience in the UK and Wales and similar to the United States (10–13). The relatively low APAPCHE II scores on admission were also in line with the data from the whole of UK and likely represent the fact that our patients were primarily admitted with single organ lung failure, as opposed to the more traditional multi-organ involvement seen in other community acquired illnesses (13). Our cohort was closely matched in terms of age, sex, comorbidities and need for assistance for daily living to the ventilated cohort of 7,425 patients in the ICNARC database (13).

Wales has a significantly lower number of critical care beds compared to England or the rest of Western Europe, a shortage which has been known for over 20 years (3, 14). Despite increasing ICU surge capacity to 9.0/100,000 population from a baseline of 4.2/100,000, and thereby reaching the pre-surge level in Lombardy, initial estimations of excess death from COVID-19 predicted that the Welsh ICU capacity could be a limiting factor in the response (2, 15).

Fortunately, the lockdown measures reduced the pressures on critical care capacity and allowed critical care units to manage beyond their normal maximal capacity, albeit with increased nurse-to-patient ratios. Importantly, we did not see any correlation between peak ICU occupancy and mortality. Despite initial concerns that critical care units may be overwhelmed with admissions, there were no ethical dilemmas of having to triage patients out of the ICU required (16). Despite the operational difficulties, RGH mortality figures compare favorably with others reported in the international literature and in the UK (10, 11, 13, 17).

At the beginning of the pandemic wave, critical care patients represented ~10–15% of the patients admitted to hospital, however after day 35, when the lockdown started to curb the number of hospital admissions, this had doubled. The reducing number of patients on the general wards enabled the redeployment of staff to the much higher work-intensity areas, including the ICU. This flexibility helped to maintain operational capabilities even when the RGH reached over 250% of conventional ICU occupancy levels (18).

The higher risk of death in the BAME group and in patients living in the most deprived areas of the country has been highlighted recently in the UK (4). The weekly analysis of the data supplied to the Intensive Care National Audit and Research Centre (ICNARC) showed that Asian/Asian British ethnic origin and living in an area at the bottom quintile of the deprivation index scale are both independently associated with mortality (19). Our results are in agreement with the findings of these reports.

Furthermore, we postulate that BAME status and social deprivation may have a synergistic effect in our population. It is known that in the UK, the BAME population is more likely to have comorbidities associated with higher susceptibility for severe COVID-19 disease such as diabetes and hypertension. One or more of these attributes were present in every patient of BAME descent in our study. Interestingly, the APACHE II scores did not differ between the Caucasian and BAME population (data not shown), further emphasizing the potential deleterious effects of the combination of chronic comorbidities and other deprivation not detected by the APACHE II score.

The risk of adverse health events, ICU admission and death are thought to be higher in people living in more deprived areas and this has been indicated in England and more recently in our study in Wales (3, 20). Interestingly international comparisons do not support this, notably in France multiple studies have shown no adverse association with low socioeconomic status and initial severity of illness on ICU admission or long-term outcome (21, 22). Currently, there is no definitive data on this subject in relation to COVID-19 disease.

Based on previously published data from England and evaluating mortality figures up to the end of April 2020, Razaq et al. proposed a risk matrix to evaluate the risk of increased vulnerability due to SARS-CoV-2 exposure in the BAME group (19). Interestingly, most of our patients admitted to the ICU were in the low risk group: aged between 30 and 69 and living in a multigenerational household.

Our data indicates that low socio-economic status in the BAME group disproportionately increases the risk of ICU admission and death from COVID-19. The exact mechanisms for this increased risk and vulnerability from COVID-19 are not clear. There has been speculation that this may be due to the higher prevalence of particular health conditions, such as cardiovascular disease or diabetes, or predisposing factors such as low vitamin D levels, among the BAME population. The findings of the OpenSAFELY collaborative, based on 17 million adult patients in England with more detailed primary care data, show that this is only a small part of the excess risk (23). Increased susceptibility for SARS-CoV-2 infection in BAME people due to genetic polymorphisms of the ACE-2 gene has also been postulated, however, this will need to be confirmed in large multi-center studies such as the one run by the ISARIC 4C Collaborators (24, 25).

Our results suggest that poorer outcomes in BAME people in COVID-19 disease may be due to exposure risks in the community, as our patients were over-represented in lower socio-economic groups. This may include higher household density with increased risk of transmission due to the lockdown, or disproportionate employment in lower band key worker roles, who either work in high exposure care environments or are unable to implement safe social distancing due to their roles, as was the case for the vast majority of our BAME patients. It is also possible that BAME status combined with low socioeconomic status delayed the presentation of patients to healthcare facilities, leading to more severe disease on admission. However, we haven't observed significant differences in the acute physiology of our patients, further pointing toward the combined chronic deleterious effect of ethnicity and deprivation.

There are significant limitations to our study: the sample size is limited and our findings might be unique to the significantly over-represented low socio-economic areas within the RGH catchment area. The small sample size also precludes more sophisticated analysis beyond simple descriptive statistics. There also might be selection bias, due to the admission policies of the ICU, however, criteria for admission into ICU have not been based on race or age. It is still possible that change in referral threshold from the other primary and secondary care providers have changed the case-mix of our population. However, comparison with the national ICNARC report suggests our case-mix is similar to elsewhere in the UK, when looking at patient factors other than socio-economic deprivation, and our findings could be extrapolated to other areas (13).

We could not compare ethnicity between hospitalized patients and those who were admitted to the critical care unit as this information has been generally poorly recorded in the hospital information system. To date there is no reliable information in the UK to corroborate our data presented here.

In conclusion we report that the first 52 patients with suspected and confirmed COVID-19 admitted to the Royal Gwent Hospital ICU has significantly stretched the critical care capacity, and it appears that the effect of the lockdown prevented the health service from being overwhelmed beyond additional capacity created in preparation for the COVID-19 pandemic. We observed a disproportionately higher admission rate and mortality rate amongst the BAME group, and it appears this may be closely linked to socio-economic deprivation which is highly prevalent in the area. Improving access to healthcare, including increasing critical care bed numbers might prevent excess mortality in this population.

## Data Availability Statement

The raw data supporting the conclusions of this article will be made available by the authors, without undue reservation.

## Ethics Statement

Ethical review and approval was not required for the study on human participants in accordance with the local legislation and institutional requirements. Written informed consent for participation was not required for this study in accordance with the national legislation and the institutional requirements.

## Author Contributions

TS: conceptualization, methodology, writing—original draft preparation, supervision, and project administration. EP and TB: investigation and data curation. TB, AD, and TS: writing—review and editing. EP and TS: visualization. All authors contributed to the article and approved the submitted version.

## Conflict of Interest

The authors declare that the research was conducted in the absence of any commercial or financial relationships that could be construed as a potential conflict of interest.

## References

[1] Huang C Wang Y Li X Ren L Zhao J Hu Y. Clinical features of patients infected with 2019 novel coronavirus in Wuhan, China. Lancet. (2020) 395:497–506. 10.1016/S0140-6736(20)30183-5 31986264 PMC7159299

[2] Grasselli G Pesenti A Cecconi M. Critical care utilization for the COVID-19 outbreak in Lombardy, Italy: early experience and forecast during an emergency response. JAMA. (2020) 323:1545–6. 10.1001/jama.2020.4031 32167538

[3] Szakmany T Preparation for SARS-CoV-2 pandemic in South Wales: practical steps. Anaesth News. (2020). Available online at: https://anaesthetists.org/Home/Resources-publications/COVID-19-guidance/Preparation-for-SARS-CoV-2-pandemic-in-South-Wales-practical-steps

[4] Szakmany T Walters AM Pugh R Battle C Berridge D Lyons R. Risk factors for 1-year mortality and hospital utilisation patterns in critical care survivors: a retrospective, observational, population-based data linkage study. Crit Care Med. (2019) 47:15–22. 10.1097/CCM.0000000000003424 30444743 PMC6330072

[5] Khunti K Singh AK Pareek M Hanif W Is ethnicity linked to incidence or outcomes of covid-19? BMJ. (2020) 369:m1548 10.1136/bmj.m1548 32312785

[6] Frawley A Hickey J Weaver C Williams J Szakmany T. Introducing a new sedation policy in a large district general hospital: before and after cohort analysis. Anaesthesiol Intens Ther. (2019) 51:4–10. 10.5603/AIT.a2019.0004 30747991

[7] Sinha P Calfee CS Cherian S Brealey D Cutler S King C. Prevalence of phenotypes of acute respiratory distress syndrome in critically ill patients with COVID-19: a prospective observational study. Lancet Respir Med. (2020). 10.1016/S2213-2600(20)30366-0. [Epub ahead of print]. 32861275 PMC7718296

[8] The RECOVERY Collaborative Group Dexamethasone in Hospitalized Patients with Covid-19 — Preliminary Report. N Engl J Med. (2020). 10.1056/NEJMoa2021436. [Epub ahead of print]. PMC7383595 32678530

[9] Pugh RJ Battle CE Thorpe C Lynch C Williams JP Campbell A. Reliability of frailty assessment in the critically ill: a multicentre prospective observational study. Anaesthesia. (2019) 74:758–64. 10.1111/anae.14596 30793278

[10] Grasselli G Greco M Zanella A Albano G Antonelli M Bellani G. Risk factors associated with mortality among patients with COVID-19 in intensive care units in Lombardy, Italy. JAMA Intern Med. (2020). 10.1001/jamainternmed.2020.3539. [Epub ahead of print]. 32667669 PMC7364371

[11] Karagiannidis C Mostert C Hentschker C Voshaar T Malzahn J Schillinger G. Case characteristics resource use, and outcomes of 10 021 patients with COVID-19 admitted to 920 German hospitals: an observational study. Lancet Respir Med. (2020) 8:853–62. 10.1016/S2213-2600(20)30316-7 32735842 PMC7386882

[12] Gupta S Hayek SS Wang W Chan L Mathews K Melamed M. Factors associated with death in critically Ill patients with coronavirus disease 2019 in the US. JAMA Intern Med. (2020). 10.1001/jamainternmed.2020.3596. [Epub ahead of print]. 32667668 PMC7364338

[13] Intensive Care National Audit and Research Centre ICNARC Report on COVID-19 in Critical Care. (2020) Available online at: www.icnarc.org/Our-Audit/Audits/Cmp/Reports (accessed July 31, 2020).

[14] Lyons RA Wareham K Hutchings HA Major E Ferguson B. Population requirement for adult critical-care beds: a prospective quantitative and qualitative study. Lancet. (2000) 355:595–8. 10.1016/S0140-6736(00)01265-4 10696978

[15] Banerjee A Pasea L Harris S Aturo GI Torralbo A Shallcross L. Estimating excess 1-year mortality associated with the COVID-19 pandemic according to underlying conditions and age: a population-based cohort study. Lancet. (2020) 395:1715–25. 10.1016/S0140-6736(20)30854-0 32405103 PMC7217641

[16] Phua J Weng L Ling L Egi M Lim CM Divatia J. Intensive care management of coronavirus disease 2019 (COVID-19): challenges and recommendations. Lancet Respir Med. (2020) 8:506–17. 10.1016/S2213-2600(20)30161-2 32272080 PMC7198848

[17] Graselli G Zangrillo A Zanella A Antonelli M Cabrini L Castelli A. Baseline characteristics and outcomes of 1591 patients infected with SARS-CoV-2 admitted to ICUs of the Lombardy Region, Italy. JAMA. (2020) 323:1574–81. 10.1001/jama.2020.5394 32250385 PMC7136855

[18] Gonzi G Gwyn R Rooney K Horner M Roy K Boktor J Our experience as Orthopaedic Registrars redeployed to the ITU emergency rota during the COVID-19 pandemic. Trans J Trauma Orthopaed Coronavirus. (2020). Available online at: https://www.boa.ac.uk/policy-engagement/journal-of-trauma-orthopaedics/journal-of-trauma-orthopaedics-and-coronavirus/our-experience-as-orthopaedic-registrars.html

[19] Centre for Evidence Based Medicine Bame COVID-19 Deaths - What Do We Know? Rapid Data & Evidence Review: 'Hidden in Plain Sight'. (2020) Available online at: www.cebm.net/wp-content/uploads/2020/05/BAME-COVID-Rapid-Data-Evidence-Review-Final-Hidden-in-Plain-Sight-compressed.pdf (accessed July 31, 2020).

[20] Welch CA Harrison DA Hutchings A Rowan K. The association between deprivation and hospital mortality for admissions to critical care units in England. J Crit Care. (2010) 25:382–90. 10.1016/j.jcrc.2009.11.003 20074907

[21] Quenot J-P Helms J Labro G Dargent A Meunier-Beillard N Ksiazek E. Influence of deprivation on initial severity and prognosis of patients admitted to the ICU: the prospective, multicentre, observational IVOIRE cohort study. Ann Intens Care. (2020) 10:20. 10.1186/s13613-020-0637-1 32048075 PMC7013026

[22] Bastian K Hollinger A Mebazaa A Azoulay E Fèliot E Chevreul K. Association of social deprivation with 1-year outcome of ICU survivors: results from the FROG-ICU study. Intens Care Med. (2018) 44:2025–37. 10.1007/s00134-018-5412-5 30353380 PMC7095041

[23] Williamson EJ Walker AJ Bhaskaran K Bacon S Bates C Morton CE. Factors associated with COVID-19-related death using OpenSAFELY. Nature. (2020) 584:430–6. 10.1038/s41586-020-2521-4 32640463 PMC7611074

[24] Lu N Yang Y Wang Y Liu Y Fu G Chen D. ACE2 gene polymorphism and essential hypertension: an updated meta-analysis involving 11,051 subjects. Mol Biol Rep. (2012) 39:6581–9. 10.1007/s11033-012-1487-1 22297693

[25] Docherty AB Harrison EM Green CA Hardwick H Pius R Norman L. Features of 20,133 UK patients in hospital with COVID-19 using the ISARIC WHO clinical characterisation protocol: prospective observational study. BMJ. (2020) 369:m1985. 10.1136/bmj.m1985 32444460 PMC7243036

